# Complexation with Ionic Polysaccharides Mitigates pH-Dependent Degradation of Soy Protein Fibril Structure and Functionality

**DOI:** 10.1021/acs.jafc.5c07627

**Published:** 2025-08-18

**Authors:** Sanjana Sawant, Audrey L. Girard

**Affiliations:** † Department of Food Science, 5228 University of Wisconsin-Madison, Madison, Wisconsin 53706, United States

**Keywords:** soy protein, fibrillation, protein−polysaccharide complexation, protein functionality, structure modification

## Abstract

This study explored complexation of soy protein fibrils with gellan gum (anionic) and chitosan (cationic) to mitigate pH-induced degradation of fibril structure and function. Unfibrillated proteins combined with polysaccharides were studied as controls. Chitosan complexation preserved fibril integrity, with a moderate particle size increase (∼2.5x) and AFM imaging of fibrils alongside some aggregates when pH was increased from 2 to 4. FTIR analysis confirmed that chitosan best preserved the fibril β-sheet structure, primarily through electrostatic interactions. A Rapid Visco Analyzer study revealed that chitosan-modified fibrils retained their gelling ability at pH 4 (final viscosity ≈ 115 cP), comparable to fibrils at pH 2 (∼93 cP). Gellan gum complexation resulted in the formation of self-supporting gels at pH 7 (final viscosity ≈ 688 cP), likely due to electrostatic repulsion between like-charged components. Overall, this work provides valuable insights into mitigating pH-induced fibril degradation, thus expanding the potential applications of protein fibrils in food matrices.

## Graphical abstract

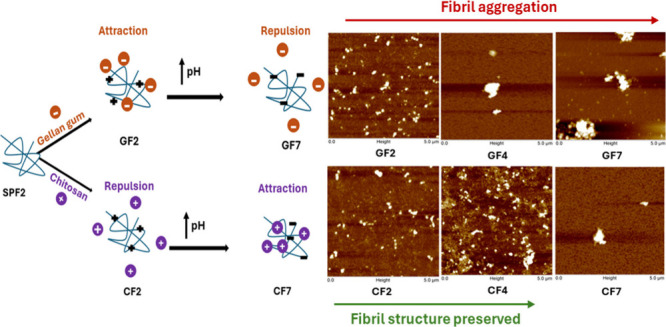

## 1 Introduction

Plant proteins are gaining significant consumer attention as sustainable and nutritious alternatives to animal proteins. Functional properties of proteins largely determine their application in different food matrices (e.g., as thickeners, emulsifiers, flavor, fat and water binding agents), and the inferior functionality of plant vs animal proteins can limit their application in the food industry.^1^ To overcome these challenges, plant protein functionality can be modified by methods such as enzymatic hydrolysis, high pressure processing, etc.^2,3^ However, modification methods often fail to reform plant proteins into well-defined, organized structures. Fibrillation with acid and heat is an emerging alternative modification route that can help achieve organized, fibrillar structures.^4,5^

Proteins that naturally exist in an unfolded state, such as muscle proteins, can easily form fibrils under physiological conditions. However, most food proteins are globular in nature and must undergo partial unfolding for exposure of aggregation-prone regions in order to form fibrils.^4^ This can be triggered by exposing the proteins to low pH (∼2) and high temperature (∼85 °C) conditions. Strong intramolecular repulsion at low pH (∼2) causes proteins to exist in a molten-globule or partially unfolded state. This can disrupt protein–protein interactions such as hydrogen bonding and hydrophobic and van der Waals interactions. Heating can further enhance this effect and promote fibril self-assembly as fibrils represent a more thermodynamically stable state compared to unfolded proteins.^4^ Resulting fibrils have excellent techno-functional abilities such as high elasticity and resistance to deformation.^5,6^ Most food systems range in pH from 4 to 7. However, it has been observed that the fibrils either become short in size or form random aggregates upon increasing pH.^6–9^ Given their immense potential for usage in food systems, it is important to develop strategies that prevent loss in fibrillar structure when the pH is increased above formation conditions (pH ≈ 2).

Complexing polysaccharides with protein fibrils can help stabilize the fibrils and enable the creation of novel food matrices that meet certain nutritional goals. However, the protein–polysaccharide complexation and resulting functionalities are greatly dependent on factors such as pH, protein–polysaccharide ratio, and ionic nature of the polysaccharides.^10,11^ Recently, a study involving complexation of ionic polysaccharides with soy protein fibrils at different pH levels found that soy protein fibrils interacted significantly with the anionic high methoxy pectin which led to improved foaming stability.^7^ Conversely, complexation with chitosan showed improved emulsifying activity due to electrostatic repulsion.^7^ However, further research is needed to understand how protein–polysaccharide interactions affect fibrils compared to unfibrillated proteins, including their structure, functionality, and the stability across different pH levels, as well as their performance under high temperature and shear conditions.

Therefore, in this study, chitosan and gellan gum were chosen as the cationic and anionic polysaccharides, respectively. Chitosan was selected, as it is the only widely accepted food-safe cationic polysaccharide currently available. Gellan gum was selected as a microbial-derived anionic polysaccharide with a low acyl variant which can reduce steric hindrance and might enhance interactions with proteins.^12^ This work focused on preserving the fibrillar structure and enabling gelation under food-relevant pH and processing conditions. While most research in this field focuses on native soy protein, this study used commercial soy protein to better assess the potential for scaling and applying the findings in industrial contexts. Hence, this study explored the complexation of soy protein fibrils synthesized from commercial soy protein with gellan gum (anionic polysaccharide) and chitosan (cationic polysaccharide) and how protein–polysaccharide interactions affected the fibril structure and function across different pH levels.

## 2 Materials and Methods

### 2.1 Materials

Soy protein isolate (≥90% protein) and low acyl gellan gum were obtained from MP Biomedicals (Santa Ana, CA) and CP Kelco (Atlanta, GA), respectively. Chitosan (degree of deacetylation ≥ 75%) was purchased from Sigma-Aldrich (St. Louis, MI). All other chemicals and reagents used were of analytical grade. Deionized (DI) water was used to prepare all of the solutions.

### 2.2 Preparation of Soy Protein Fibrils

A 3.5% (w/w) soy protein isolate (SP) dispersion was prepared in DI water, and the pH was adjusted to 2 using 6 M HCl. This dispersion was heated at 85 °C for 16 h with continuous stirring (300 rpm) to induce fibrillation, followed by immediate cooling in an ice bath.^9^ The pH of the unfibrillated and fibrillated dispersions was adjusted to 2, 4, and 7 using 6 M HCl or 5 M NaOH, a standard approach in pH-shifting studies to have negligible impact on ionic strength.^13,14^ This resulted in samples SP2, SP4, and SP7 and SPF2, SPF4, and SPF7, respectively.

### 2.3 Addition of Polysaccharides and Complexation Formation

Gellan gum and chitosan were added to the protein dispersions (SP2 and SPF2) to achieve a polysaccharide concentration of 0.3%, a value selected based on preliminary trials and existing literature to allow comparability. The samples were then stirred for 2 h at 800 rpm to ensure uniform dispersion, after which the pH of the samples was adjusted, as mentioned in Section 2.2. The samples were stirred for 4 h at 800 rpm to allow uniform dispersion and protein–polysaccharide complexation. Unfibrillated and fibrillated samples with gellan gum and chitosan at pH 2, 4, and 7 are referred to as GG2, GG4, GG7, GF2, GF4, and GF7 and CT2, CT4, CT7, CF2, CF4, and CF7, respectively.

### 2.4 Particle Size and ζ-Potential

Particle size and ζ-potential were measured to understand the effects of pH and polysaccharide addition on the size and stability of the fibrils. Samples were diluted 100-fold with DI water at the corresponding pH. The particle size and ζ-potential were determined by using the Brookhaven 90Plus analyzer (Brookhaven Instruments Corporation, New Hampshire, USA). Particle size and ζ-potential measurements were conducted for the fibrillated samples only. The presence of large aggregates in the unfibrillated samples made accurate measurements unfeasible due to instrumentation limitations.

### 2.5 Thioflavin T (ThT) Assay

The ThT assay was performed according to the method of Wan and Guo.^9^ ThT stock solution was made by dispersing 8 mg of ThT into 10 mL of 10 mM phosphate buffer (pH 7.0) containing 50 mM NaCl. This stock solution was filtered with a 0.22 μm syringe filter to remove undissolved ThT. The working solution was made by diluting the stock solution 50 times. Then, 100 μL of the sample was mixed with 4 mL of ThT working solution and reacted for 2 min. The samples were excited at a wavelength of 440 nm, and fluorescence emission was measured at 482 nm using the Biotek Synergy H1 multimode microplate reader (Biotek, Vermont, USA).

### 2.6 Surface Hydrophobicity (H_0_)

Surface hydrophobicity was determined using the ANS method. Four milliliters of protein dispersions (0.02–0.1 mg/mL) at the corresponding pH were mixed with 40 μL of 8 mM ANS and incubated in the dark for 10 min. Fluorescence was measured at 390 nm excitation and 470 nm emission wavelengths using a Biotek Synergy H1 plate reader (Biotek, Vermont, USA). H_0_ was calculated as the slope of fluorescence intensity versus protein concentration.

### 2.7 Sodium Dodecyl Sulfate–Polyacrylamide Gel Electrophoresis (SDS-PAGE)

SDS-PAGE was carried out to study the effects of fibrillation and the addition of polysaccharides on the protein molecular weight (MW) profile. Samples were diluted with sample buffer and heated at 95 °C for 5 min to solubilize the proteins. Ten microliters of the diluted samples were loaded into the wells of the Mini-PROTEAN TGX stain-free precast gel (12%, Bio-Rad Laboratories). The electrophoresis was performed at 200 V for 35 min using a Power PAC 3000 (Bio-Rad Laboratories, Hercules, CA). The gels were then imaged using a ChemiDoc Imaging System (Bio-Rad Laboratories, Hercules, CA).^15^

### 2.8 Attenuated Total Reflectance Fourier-Transform Infrared (ATR-FTIR) Spectroscopy

FTIR spectra of the samples were analyzed to study the effects of fibrillation on the protein secondary structure. Spectral information on freeze-dried samples was recorded in the wavelength range of 4000–500 cm^–1^ (resolution: 2 cm^–1^) using a Nicolet IS50 FTIR spectrometer (Thermo Nicolet, Ltd., Waltham, MA, USA) equipped with a Pike IRIS diamond ATR (PIKE Technologies, Wisconsin, USA). All spectra were smoothed using the Savitzky–Golay function, and baseline correction was performed using Origin 2023 software. Curve fitting of the amide I region was performed using Microsoft Excel Solver with Gaussian functions fitted to individual peaks for secondary structure analysis.^16,17^

### 2.9 Atomic Force Microscopy (AFM)

AFM of the samples was performed to study the effects of pH and ionic polysaccharides on the morphology of the fibrils. A 10 μL drop of the sample was (1) placed on a freshly cleaved mica disc mounted on a glass slide, (2) allowed to sit for 2 min, (3) rinsed with DI water at the corresponding pH, and (4) dried with compressed air. The samples were imaged using a Bioscope Catalyst AFM instrument (Bruker, Santa Barbara, CA) in tapping mode. A silicon and triangle tip on the nitride lever was used. Images were processed and analyzed with NanoScope Analysis software version 1.40 (Bruker, Santa Barbara, CA).^15,18^

### 2.10 Scanning Electron Microscopy (SEM)

SEM was carried out to visualize the surface characteristics of the aggregates formed. Samples were diluted 1000-fold by using DI water at the corresponding pH. A 10 μL portion of the diluted sample was deposited on carbon adhesive tabs glued on sample holders and air-dried overnight. The samples were sputter-coated with a 2 nm platinum layer using the Leica ACE600 (Leica Microsystems, Wetzlar, Germany) and imaged with a Zeiss Gemini 450 FESEM (Carl Zeiss AG, Oberkochen, Germany) at an accelerating voltage of 3 kV.^19^ Image processing was performed using ImageJ software.

### 2.11 Molecular Interactions Based on Protein Solubility in Various Reagents

Six reagents were used to solubilize and extract proteins from the samples to study molecular interactions involved in fibrillation and protein–polysaccharide complexation.^1^ The reagents were as follows: (A) DI water at pH 2, 4, 7; (B) 0.05 M NaCl solution at pH 2, 4, 7; (C) 1 M thiourea solution at pH 2, 4, 7; (D) 4 M urea solution at pH 2, 4, 7; (E) 50 mM DTT solution at pH 2, 4, 7; (F) 4 M urea and 50 mM DTT solution at pH 2, 4, 7. NaCl disrupts the electrostatic interactions. Thiourea primarily disrupts the hydrophobic interactions. Urea mainly targets hydrogen bonding in proteins. DTT targets covalent bonds. The combination of urea and DTT targets both hydrogen and disulfide bonds.

Proteins were solubilized in the reagents at room temperature following the method described by Sawant et al.^3^ with minor modifications. Briefly, 100 μL of the samples were added to 1 mL of each solvent and vortexed for 1 h. The solvents were centrifuged at 10,000*g* for 20 min, and supernatants were collected. The solubilized protein content of the supernatants was determined by using a commercial Coomassie protein assay kit with BSA as a standard. The solubilized protein was calculated and expressed as a percentage of the total protein present in the samples. To emphasize the independent effects of the reagents on molecular interactions (i.e., without the effect of DI water on solubility), the solubilized protein content for DI water was subtracted from the solubilized protein content of the reagents.

### 2.12 Rapid Visco Analysis (RVA)

The samples were analyzed using the Rapid Visco Analyzer (RVA 4800; PerkinElmer, USA) to observe viscosity trends indicative of gelation under heat and shear conditions. The testing protocol consisted of five distinct phases. The RVA temperature program was as follows: 0.0–1.0 min at 50 °C; 1.0–7.4 min ramping to 130 °C; 7.4–10.10 min hold at 130 °C; 10.10–18.55 min cooling to 25 °C; and 18.55–30 min hold at 25 °C. The paddle speed was set to 960 rpm for the first 10 s, then reduced to 160 rpm until 18.55 min, at which point it was stopped (0 rpm) and samples were held steady for 5 min to ‘set’. Shear at 160 rpm was reintroduced at 24 min to assess gel stability, with viscosity changes being used to indicate potential gel collapse.

### 2.13 Phase Stability

To study the effects of pH, polysaccharide addition, and RVA treatment on phase separation, 15 mL of each sample before and after RVA treatment was stored in glass vials in refrigerated conditions overnight. Samples were observed visually.

### 2.14 Statistical Analysis

A one-way ANOVA followed by a Tukey Kramer HSD test was performed to assess significant differences among groups using JMP Pro 17 Software (SAS) with a significance level of *p* < 0.05. All tests were performed in triplicate.

## 3 Results and Discussion

### 3.1 Effects of pH and Polysaccharide Addition on Fibril Particle Size and Zeta Potential

Samples were analyzed for their particle size and zeta potential with dynamic light scattering to identify the effects of pH and polysaccharide addition on protein fibrils. Fibrillated soy protein samples at pH 2 (SPF2) exhibited the smallest particle size and the second-highest zeta potential (Figure 1), indicating a good phase stability.^7^ Particle size of the SPF sample significantly increased upon changing the pH to 4 accompanied by a marked decrease in zeta potential likely due to aggregation near the isoelectric point of soy proteins (pI ≈ 4.5).^9^ At pH 7, the particle size decreased relative to pH 4 but remained greater than at pH 2. This may be because pH 7 is further from the pI as compared to pH 4, leading to reduced aggregation of the proteins.^9^ The zeta potential of SPF7 was lower than that of SPF2 but higher than that of SPF4, consistent with the particle size trends. The addition of ionic polysaccharides to the SPF2 samples (GF2 and CF2) did not significantly affect particle size (Figure 1A). However, GF2 showed a lower zeta potential than SPF2 and CF2 (Figure 1B), likely due to associative electrostatic attraction between the anionic gellan gum and the positively charged protein, forming coacervates.^7,11^

**Figure 1:**
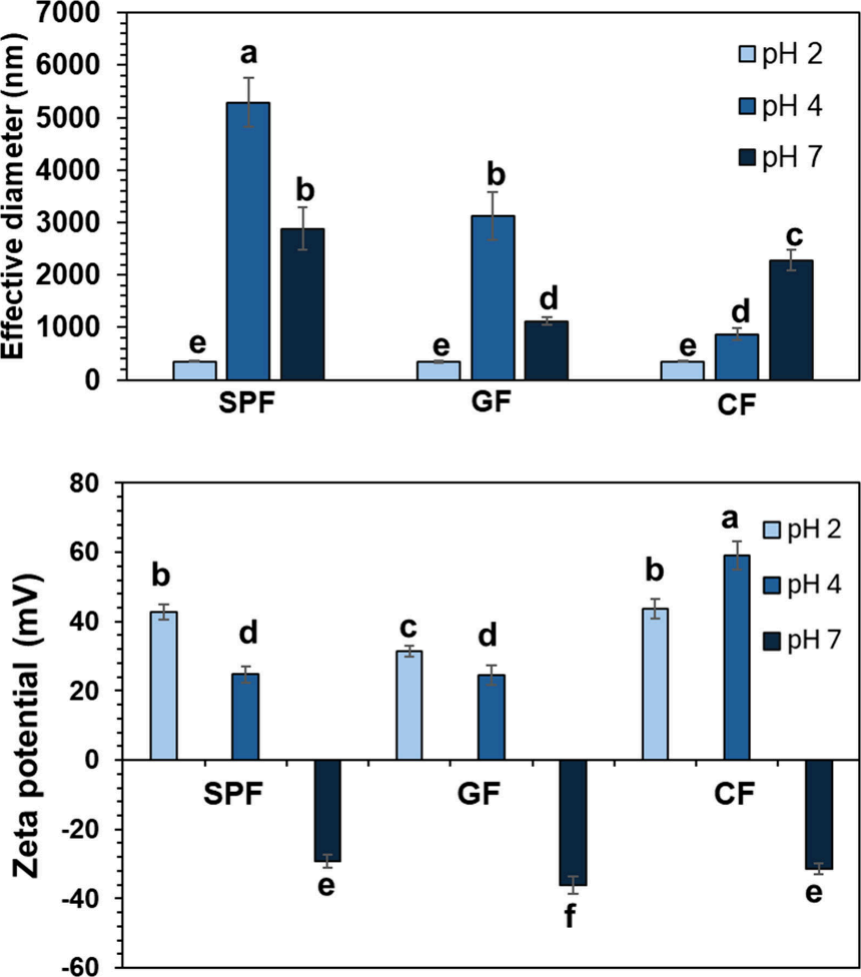
(A) Particle size and (B) ζ-potential of fibrillated protein samples analyzed using dynamic light scattering. SPF = fibrillated soy protein; GF = fibrillated soy protein with gellan gum; CF = fibrillated soy protein with chitosan. Letters denote significant differences across all samples (*p* < 0.05).

GF samples showed a trend in particle size and zeta potential similar to that of SPF samples upon pH adjustment. At pH 7, since both soy protein and gellan gum were negatively charged, the composite system exhibited higher negative zeta potential values compared to SPF7 and CF7 (Figure 1B), indicating electrostatic repulsion and dissociation of the gellan gum–fibril complex. However, residual molecular interactions (e.g., van der Waals, hydrogen bonding, and hydrophobic interactions) could prevent complete dissociation of the complex and inhibit protein aggregation.^20^ As a result, GF4 and GF7 had smaller particle sizes than their SPF counterparts, suggesting that gellan gum might relatively inhibit pH-induced aggregation.

Chitosan has a p*K*_a_ of ∼6.5 and remains in a protonated state below this value. Thus, at pH 2, both chitosan and the protein are positively charged, leading to an increased electrostatic repulsion, as reflected by the high zeta potential value observed (Figure 1B). For CF samples, the high zeta potential values observed at pH 2 indicate electrostatic repulsion between cationic chitosan and positively charged soy protein (Figure 1B). Thus, the biopolymers might not form a complex, but rather remain evenly distributed through the matrix as a single-phase solution due to steric hindrance and electrostatically driven phase separation occurring at a microscopic level.^11^ The CF4 sample showed the smallest particle size compared to all other samples at pH 4 (Figure 1A). It also showed the highest zeta potential values, even higher than those of the SPF2 samples. This indicates a strong electrostatic repulsion which could help inhibit protein–protein interactions and aggregation observed in the SPF samples.^9^ An increase in particle size and a decrease in zeta potential upon changing the pH to 7 was observed. Although pH 7 is above its p*K*_a_, chitosan does not fully deprotonate at this pH level.^21,22^ This can enable electrostatic attraction with the negatively charged proteins at pH 7 leading to formation of larger coacervates or precipitates.^8,11^

### 3.2 Effects of pH Change and Polysaccharide Addition on Fibril Surface Hydrophobicity (H_0_)

The distribution of hydrophobic regions along the protein fibril surfaces was quantified with ANS, a fluorescent probe that binds to protein hydrophobic regions.^23^ Fibrillated samples at pH 2 and 4 showed increased surface hydrophobicity compared to the unfibrillated samples regardless of polysaccharide addition (Figure 2). This indicates that heating the soy proteins in acidic conditions for prolonged periods caused unfolding and exposure of hydrophobic groups.^23^ Surface hydrophobicity decreased for SP, SPF, GG, and GF samples upon increasing the pH to 4. This likely occurred because of lowered electrostatic stability (Figure 1B) and increased protein–protein interactions promoting aggregation and burial of hydrophobic regions, thus leading to a reduction in ANS binding.^24^ H_0_ decreased significantly at pH 7 for all samples due to exposure of polar groups.^7^

**Figure 2:**
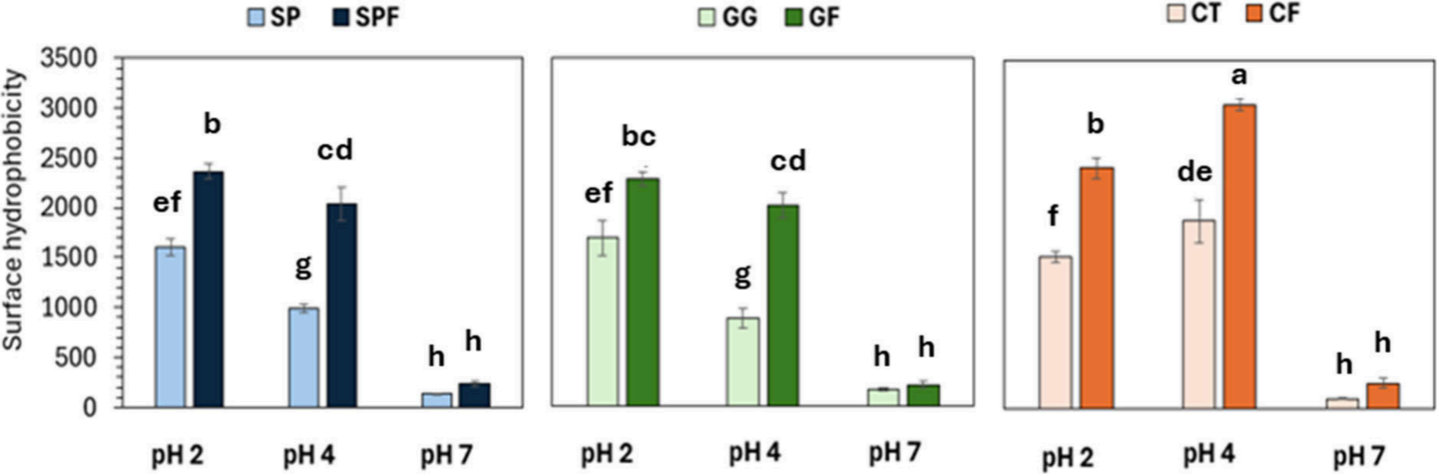
Surface hydrophobicity (H_0_) of soy protein samples quantified using the hydrophobic fluorescent probe, ANS. SP = unfibrillated soy protein; GG = unfibrillated soy protein with gellan gum; CT = unfibrillated soy protein with chitosan; SPF = fibrillated soy protein; GF = fibrillated soy protein with gellan gum; CF = fibrillated soy protein with chitosan. Letters denote significant differences across all samples (*p* < 0.05).

For CT and CF samples, the surface hydrophobicity increased at pH 4 (Figure 2). This increase likely occurred either because of conformational changes in the protein due to increased interactions with chitosan or because the negatively charged ANS interacted more strongly with a positively charged matrix (Figure 1B).^7^ Apart from the samples with chitosan at pH 4, polysaccharide addition did not have a significant impact on H_0_. The strong effect of pH on the protein structure may have masked any minor effects induced by polysaccharide addition.

### 3.3 Effects of Fibrillation on Protein Profiles in SDS-PAGE

The unfibrillated soy protein samples showed an electrophoretic pattern typical of soy proteins (Figure 3), with bands observed for α′ (∼72 kDa), α (∼68 kDa), and β (∼53 kDa) subunits in β-conglycinin (7S) and acidic (∼29–33 kDa) and basic (∼18–22 kDa) subunits in glycinin (11S).^5^ No significant differences were observed with polysaccharide addition or pH, likely due to the noncovalent nature of interactions. However, lanes for unfibrillated samples at pH 2 appeared darker, possibly due to Coomassie Brilliant Blue’s greater affinity to positively charged proteins under acidic conditions (Figure 1B), as the dye can interact strongly via its negatively charged sulfonic groups with positive charges on the protein surface.^25,26^ Consequently, these lanes may have required more time to destain compared to those at a higher pH.

**Figure 3:**
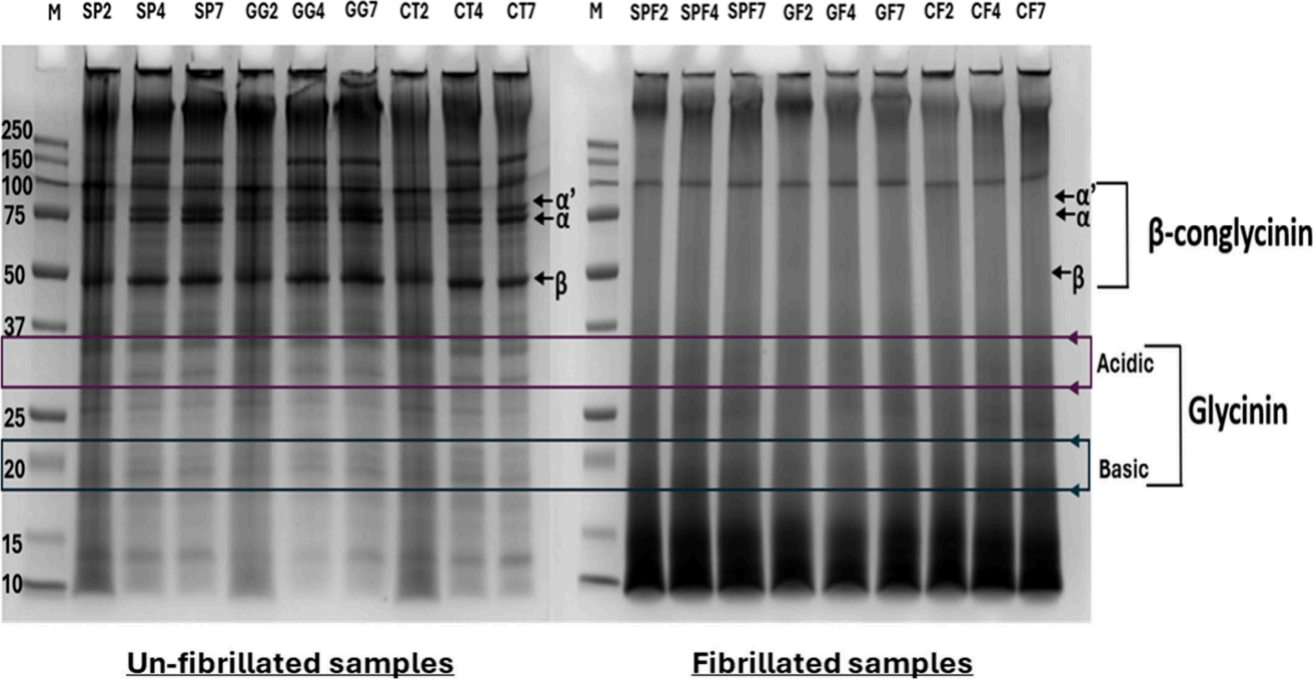
SDS-PAGE profiles of different samples separated under nonreducing conditions. Lanes are labeled by sample code: M = molecular weight marker; SP2/4/7 = unfibrillated soy protein at pH 2, 4, or 7; GG2/4/7 = unfibrillated soy protein with gellan gum at pH 2, 4, or 7; CT2/4/7 = unfibrillated soy protein with chitosan at pH 2, 4, or 7; SPF2/4/7 = fibrillated soy protein at pH 2, 4, or 7; GF2/4/7 = fibrillated soy protein with gellan gum at pH 2, 4, or 7; CF2/4/7 = fibrillated soy protein with chitosan at pH 2, 4, or 7, respectively. The bands representing the subunits of β-conglycinin (7S) and glycinin (11S) soy globulins are labeled on the right side of the electrophoretogram.

Upon fibrillation, most bands above 37 kDa disappeared, except for some high molecular weight aggregates (>250 kDa), which likely remained insoluble even in nonreducing SDS-PAGE conditions. Their insolubility could have hindered their conversion into fibrils, which are more readily formed from soluble protein fractions.^27,28^ A band at ∼100 kDa, likely lipoxygenase, was observed despite fibrillation. Munialo et al.^29^ observed a similar band which disappeared between 10 and 22 h of heating. Lower MW bands were very faint, with dense smears below ∼18 kDa (Figure 3) indicating hydrolysis of proteins due to prolonged heating at a low pH for fibrillation.^5,30^ Specifically, bands representing β-conglycinin subunits were not visible in fibrillated samples, indicating their involvement in fibril formation. The faint glycinin subunit bands observed even after fibrillation indicated their relatively limited involvement. Thus, β-conglycinin was more readily hydrolyzed during fibrillation as compared to glycinin.^30^

### 3.4 Effects of pH and Polysaccharide Addition on Fibril Structure

The ThT assay was used to detect β-sheet structures in protein fibrils, with higher fluorescence intensity indicating more β-sheets.^31^ Unfibrillated samples showed no significant difference in fluorescence intensity regardless of pH or polysaccharide addition (Figure 4), indicating that these factors alone do not significantly affect the existing β-sheet content. SPF2 exhibited a 2x increase in fluorescence intensity compared to SP2 (Figure 4), confirming fibril formation. However, fluorescence intensity dropped significantly upon increasing the pH to 4, likely due to aggregation near the isoelectric point (pI).^7,9^ There was no significant difference in ThT fluorescence intensity between the SPF4 and SPF7 samples (Figure 4), indicating that maximum degradation occurred upon passing through the pI.

**Figure 4:**
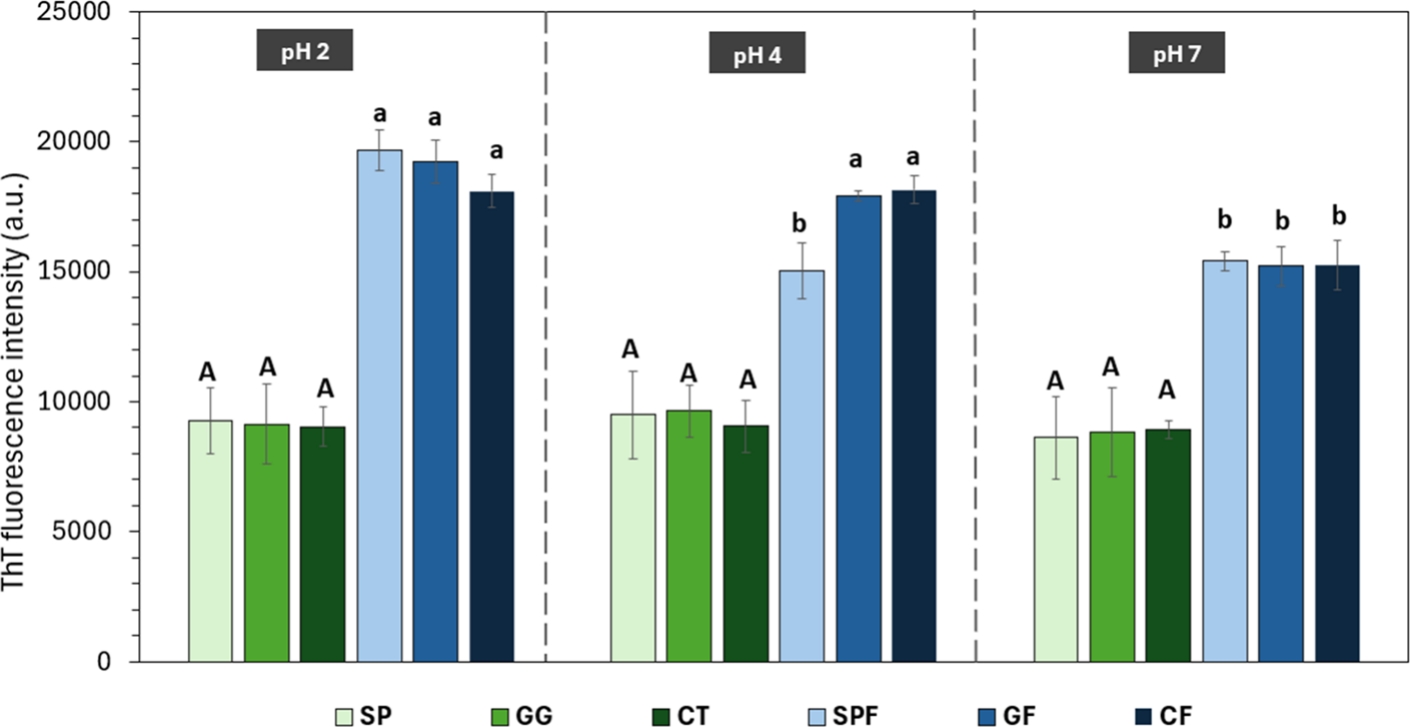
ThT fluorescence spectroscopy of soy protein samples modified with fibrillation and polysaccharide addition under different pH conditions. SP = unfibrillated soy protein; GG = unfibrillated soy protein with gellan gum; CT = unfibrillated soy protein with chitosan; SPF = fibrillated soy protein; GF = fibrillated soy protein with gellan gum; CF = fibrillated soy protein with chitosan. Uppercase and lowercase letters denote significant differences (*p* < 0.05) across the unfibrillated and fibrillated samples, respectively.

Polysaccharide addition did not significantly alter the fluorescence at pH 2 (Figure 4). For CF4, electrostatic repulsion may have prevented formation of amorphous aggregates, thus limiting the loss of fluorescence intensity.^7,32^ But for CF7, reduced fluorescence could be explained by aggregation and structural changes resulting from electrostatic attraction leading to formation of larger coacervates.^32^ Despite low zeta potential and high particle size (Figure 1), GF4 showed similar fluorescence intensity as the SPF2, GF2, CF2, and CF4 (Figure 4), possibly due to positively charged ThT’s affinity for anionic groups.^7^ This can also be applicable for GF7, given that the number of negative groups on the surface of the proteins can increase at pH 7.^10^ Regardless, a relative decrease in fluorescence intensity as compared to GF2 and GF4 was observed for GF7 likely due to electrostatic repulsion and depletion interactions.^33^

### 3.5 Secondary Structure Analysis of Protein Fibrils Using ATR-FTIR

Due to possible interference from protein aggregation in ThT binding, ATR-FTIR was used to further analyze β-sheet structures. All samples showed an amide I band (1700–1600 cm^–1^), an amide II band (1575–1480 cm^–1^), and an amide A band (3200–3400 cm^–1^) (Figure S1). Since the amide I region is more sensitive to changes in protein secondary structures, peak fitting of this region was done to analyze changes due to fibrillation, ionic polysaccharide addition, and pH adjustment. The total β-sheet content of the SP2 samples increased from 60.7% to 62.7% upon undergoing fibrillation (Table 1). However, β-sheets can be further distinguished into parallel (∼1633 cm^–1^) and antiparallel β-sheets (∼1618 and ∼1680 cm^–1^).^5,34^ SP2 and SPF2 had similar parallel β-sheet contents, but SPF2 had a significantly higher antiparallel β-sheet content. Moreover, the increased antiparallel β-sheet structure was accompanied by decreased α-helix and random coil structures (∼1649 cm^–1^), suggesting structure conversion commonly observed during fibril formation.^5,35^ A higher relative contribution of antiparallel β-sheets at 1618 cm^–1^ was observed as compared to 1680 cm^–1^. This is consistent for fibril structure and indicates strong hydrogen bonding, whereas absorbance at 1680 cm^–1^ reflects weaker hydrogen bonds and dipole–dipole interactions.^35–37^

**Table 1:** FTIR Peak Fitting of the Amide I Region (1600–1700 cm^–1^) of Soy Protein Samples

sample^a^	antiparallel β-sheets (∼1618 cm^–1^)^b^	parallel β-sheets (∼1633 cm^–1^)	α-helix and random coil (∼1649 cm^–1^)	β-turns (∼1665 cm^–1^)	antiparallel β-sheets (∼1680 cm^–1^)
SP2	19.51 ± 0.61^b^	34.28 ± 1.31^cdef^	26.82 ± 0.29^def^	12.47 ± 0.14^bcde^	6.92 ± 0.58^c^
SPF2	24.54 ± 1.36^a^	33.13 ± 0.64^defg^	23.84 ± 1.02^g^	13.47 ± 0.39^ab^	5.02 ± 1.35^d^
SP4	17.28 ± 0.55^bc^	34.94 ± 0.29^abcde^	26.98 ± 0.55^cdef^	12.00 ± 0.30^def^	8.80 ± 0.33^b^
SPF4	13.97 ± 1.57^def^	36.04 ± 1.23^abcd^	27.75 ± 0.62^bcdef^	12.81 ± 0.31^bcde^	9.43 ± 0.66^ab^
SP7	11.38 ± 0.48^f^	37.94 ± 1.17^a^	27.79 ± 0.85^bcdef^	12.85 ± 0.18^bcde^	10.04 ± 0.29^ab^
SPF7	12.90 ± 1.90^def^	35.31 ± 1.78^abcd^	27.95 ± 0.71^bcde^	13.28 ± 0.37^abc^	10.56 ± 0.10^a^
GG2	18.46 ± 0.91^b^	34.43 ± 1.52^bcdef^	28.12 ± 1.35^bcde^	12.00 ± 0.48^def^	6.99 ± 0.15^c^
GF2	23.02 ± 0.36^a^	30.26 ± 1.02^g^	26.53 ± 0.67^efg^	13.45 ± 0.56^ab^	6.73 ± 0.83^c^
GG4	15.44 ± 0.92^cd^	35.94 ± 0.01^abcd^	27.04 ± 1.21^cdef^	12.20 ± 0.48^cdef^	9.37 ± 0.28^ab^
GF4	19.79 ± 0.40^b^	32.07 ± 0.94^efg^	25.77 ± 0.59^efg^	13.49 ± 0.43^ab^	8.88 ± 0.36^ab^
GG7	12.70 ± 0.48^ef^	37.51 ± 1.34^ab^	26.95 ± 1.13^cdef^	12.84 ± 0.45^bcde^	10.00 ± 0.08^ab^
GF7	19.63 ± 1.39^b^	24.77 ± 1.16^h^	31.19 ± 1.68^a^	14.26 ± 0.82^a^	10.16 ± 1.09^ab^
CT2	18.72 ± 0.10^b^	33.83 ± 0.31^def^	29.73 ± 0.60^abc^	11.00 ± 0.16^f^	6.72 ± 0.25^c^
CF2	24.14 ± 0.51^a^	32.06 ± 0.53^efg^	25.15 ± 0.66^fg^	13.23 ± 0.21^abc^	5.41 ± 0.15^cd^
CT4	14.17 ± 0.17^de^	35.45 ± 0.84^abcd^	29.60 ± 0.86^abcd^	11.78 ± 0.26^ef^	9.00 ± 0.12^ab^
CF4	19.66 ± 0.60^b^	31.38 ± 0.75^fg^	26.61 ± 0.57^efg^	13.03 ± 0.53^bcd^	9.32 ± 0.17^ab^
CT7	11.81 ± 0.28^ef^	37.38 ± 1.07^abc^	27.65 ± 1.26^bcdef^	12.68 ± 0.25^bcde^	10.49 ± 0.54^a^
CF7	12.30 ± 0.48^ef^	33.74 ± 0.67^def^	29.97 ± 0.59^ab^	13.44 ± 0.08^ab^	10.54 ± 0.32^a^

a Unfibrillated soy protein at pH 2, 4, 7 = SP2, 4, 7; unfibrillated soy protein with gellan gum at pH 2, 4, 7 = GG2, 4, 7; unfibrillated soy protein with chitosan at pH 2, 4, 7 = CT2, 4, 7, respectively. Fibrillated soy protein at pH 2, 4, 7 = SPF2, 4, 7; fibrillated soy protein with gellan gum at pH 2, 4, 7 = GF2, 4, 7; fibrillated soy protein with chitosan at pH 2, 4, 7 = CF2, 4, 7, respectively.

b Letters denote significant differences in samples within the same secondary structure category (*p* < 0.05).

Increasing the pH of SPF2 led to a decrease in antiparallel β-sheets (∼1618 and ∼1680 cm^–1^) and an increase in α-helix and random coil content (Table 1), indicating loss of fibril structure and conformation. This likely occurs near the protein’s pI (∼4.5), where loss of charge promoted aggregation.^8^ Similar to ThT results (Figure 4), no significant difference was observed between antiparallel β-sheet content of SPF4 and SPF7, confirming maximum degradation near the pI. Polysaccharide addition (GF2, CF2) did not significantly alter secondary structure at pH 2 (Table 1). At pH 4, both GF and CF samples showed decreased antiparallel β-sheet structures at ∼1618 cm^–1^ and increased structures at ∼1680 cm^–1^; however, unlike the SPF samples, total antiparallel β-sheet content (∼1618 and ∼1680 cm^–1^) did not decrease and there was no increase in α-helix and random coil content. Thus, for GF4 and CF4, the β-sheets did not undergo major structural rearrangement, and any observed changes were likely caused by aggregation and weakening of hydrogen bonds.^9^

At pH 7, GF and CF showed different trends. In GF7, total antiparallel β-sheet content did not decrease (Table 1); instead, parallel β-sheets decreased while α-helix and random coil increased. Hydrogen bonding weakened compared to GF2, though not significantly different from GF4. The exact mechanism of this and its selectivity for parallel β-sheets remain unknown and can be explored in future studies. For the CF7, antiparallel β-sheets (specifically at ∼1618 cm^–1^) decreased along with an increase in α-helix and random coil content indicating loss of typical fibril structure.^9^ Strong electrostatic attraction leading to complex coacervation at pH 7 possibly contributed to this.^32^ Future studies can build upon the structural insights provided by FTIR through the use of advanced methods such as nuclear magnetic resonance (NMR) or X-ray diffraction (XRD) to achieve greater molecular-level resolution.

### 3.6 Molecular Interactions Involved in Fibril Formation and Polysaccharide Complexation

To study specific molecular interactions involved in fibril formation and complexation with polysaccharides, protein solubility of the samples was measured in different reagent combinations, including DI water, NaCl, thiourea, urea, and dithiothreitol (DTT). Fibrillated samples at pH 2 (SPF2, GF2, CF2) showed ∼2.5x higher solubility in DI water than unfibrillated samples (Figure 5A), likely due to structural changes caused by fibrillation. For other reagents, DI water solubility was subtracted to highlight their independent effect. Solubility in NaCl was close to zero for all samples, suggesting that electrostatic forces were not the primary stabilizing forces for the fibrils or complexes at pH 2. Unfibrillated samples were poorly soluble in DI water but dissolved well in urea and thiourea, indicating that breaking hydrogen bonds and hydrophobic interactions, respectively, alleviated aggregation.^38,39^ Fibrillated samples were most soluble in urea and then thiourea, showing that hydrogen bonding followed by hydrophobic interactions were key interactions involved in fibril formation. Fitzpatrick et al.^40^ also showed that amyloid fibrils are primarily stabilized by hydrogen bonding. Solubility with DTT (alone or with urea) was low, suggesting disulfide bonds were not strongly involved, likely because cysteine residues are protonated during fibrillation.^41^

**Figure 5:**
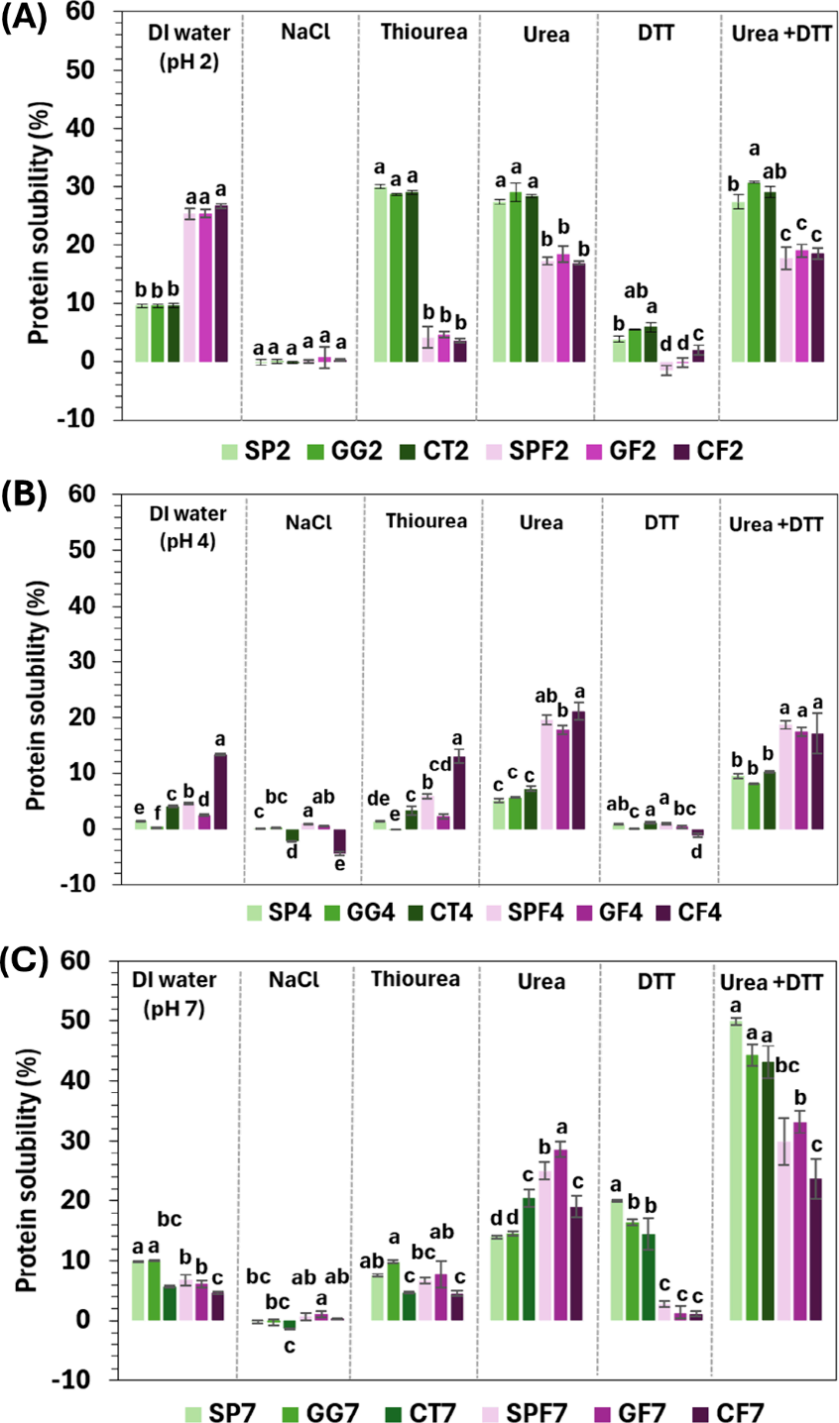
Molecular interactions studied by solubilizing samples in different reagents. (A) Samples at pH 2; (B) samples at pH 4; and (C) samples at pH 7. Unfibrillated soy protein at pH 2, 4, 7 = SP2/4/7; unfibrillated soy protein with gellan gum at pH 2, 4, 7 = GG2/4/7; unfibrillated soy protein with chitosan at pH 2, 4, 7 = CT2/4/7, respectively. Fibrillated soy protein at pH 2, 4, 7 = SPF2/4/7; fibrillated soy protein with gellan gum at pH 2, 4, 7 = GF2/4/7; fibrillated soy protein with chitosan at pH 2, 4, 7 = CF2/4/7, respectively. Letters denote significant differences between samples at the same pH treated with the same reagent (*p* < 0.05).

Samples at pH 4 were less soluble in DI water than those at pH 2, likely because pH 4 is closer to the pI (Figure 5B). However, CF4 had the highest solubility in DI water due to electrostatic repulsion, which helped preserve fibril characteristics. Upon extraction with NaCl, the solubility of CF4 (and then CT4) decreased compared to DI water. This suggests that NaCl may have disrupted electrostatic interactions that were stabilizing the fibril structure at pH 4. Anvar et al.^38^ and Dent et al.^39^ also found that breaking hydrophobic interactions with thiourea lowered protein solubility in zein and chickpea hydrolysates due to destabilization of hydrophobic-effect-mediated protein solubility. CF4 also showed higher solubility in both thiourea and urea, suggesting the involvement of hydrogen bonding and hydrophobic interactions—both linked to fibril formation.^31^ Additionally, while both chitosan and soy proteins are positively charged at pH 2–4 causing electrostatic repulsion between the two, associative interactions can still occur due to localized charge interactions and hydrophobic effects, resulting in a functional association even in the absence of strong electrostatic attraction.^32^ Lastly, as at pH 2, disulfide bonds did not play a major role in any sample.

At pH 7, fibrillated samples showed decreased solubility in DI water as compared to unfibrillated samples (Figure 5C). This could be due to the loss of fibrillar structure and increased aggregation. GF7 exhibited the highest solubility in urea, suggesting that a substantial portion of the hydrogen bonding is likely stabilizing aggregates formed due to the pH change and electrostatic repulsion between gellan gum and soy protein. Molecular interactions in the unfibrillated samples were primarily governed by disulfide bonds, as evidenced by their solubility being highest in DTT. However, this trend was not observed in the fibrillated samples. Fibril formation involves the burial of free −SH groups, and subsequent aggregation due to pH changes may further prevent the exposure of these groups, leading to reduced reactivity and solubility.^42,43^

### 3.7 Effects of pH and Polysaccharides on Morphology of the Fibrillar Aggregates

AFM and SEM were used to visualize the aggregates. AFM images of SP2 (Figure S2) showed random aggregates, while SPF2 samples (Figure 6) showed smaller, curved fibrils alongside long, thin fibrils and some amorphous aggregates. Previous work showed that <50% of treated proteins form fibrils, while the rest form peptides or amorphous protein aggregates.^29^ No fibrils were observed in SPF4 and SPF7 samples, indicating a loss of fibril structure upon pH adjustment. Both SP (Figure S2) and SPF (Figure 6) samples showed larger aggregates at pH 4 due to proximity to the pI.^8,9^ At pH 7, the aggregate size decreased compared to that at pH 4. However, unlike earlier reports,^8,9^ fibril remnants did not reappear at pH 7, suggesting irreversible damage upon passing through the pI (∼4.5).

**Figure 6:**
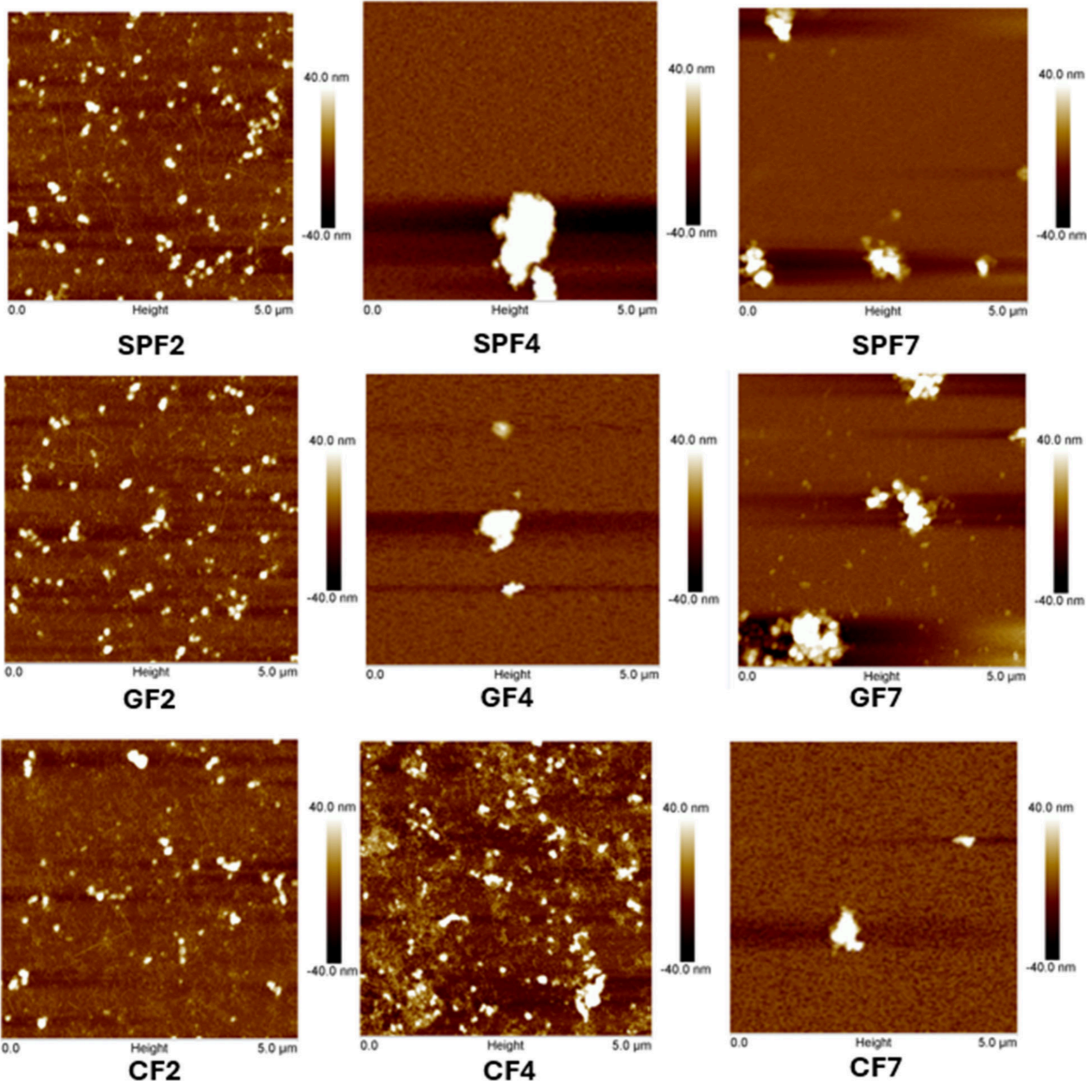
AFM images (5 × 5 μm) of fibrillated samples with and without addition of polysaccharides. Fibrillated soy protein at pH 2, 4, 7 = SPF2/4/7; fibrillated soy protein with gellan gum at pH 2, 4, 7 = GF2/4/7; fibrillated soy protein with chitosan at pH 2, 4, 7 = CF2/4/7, respectively.

Similar to particle size observations (Section 3.1), the addition of gellan gum or chitosan did not affect fibril morphology at pH 2 (Figure 6). Adjusting the pH to 4 and 7 increased aggregation and reduced fibrillar features in GF4 and GF7. In the case of GF7, the antiparallel β-sheet structure was relatively preserved as compared with SPF7 samples (Section 3.5, Table 1), suggesting that the fibril morphology should have also been maintained. However, AFM revealed the presence of aggregates rather than distinct fibrils. This could be attributed to a combination of increased hydrogen bonding and electrostatic repulsion (Section 3.7, Figure 5C) between the anionic gellan gum and the negatively charged protein at pH 7. These interactions may promote physical entanglement, resulting in a dual-network structure.^44^ This is unlike typical fibril aggregation, as it might obscure fibrillar morphology while still preserving the underlying β-sheet structure and associated functional properties. In contrast, CF4 showed fibrils and resisted formation of large aggregates, likely due to stronger electrostatic repulsion between chitosan and soy protein at pH 4 (Figure 1B).^7,8,32^ Some aggregation between the fibrils in CF4 sample was observed, which could be attributed to lateral associations between fibrils closer to the pI.^9^ At pH 7, CF7 showed large coacervates likely due to electrostatic attraction between oppositely charged biopolymers.^11,32^

SEM was used to visualize these aggregated surface features. SPF2 samples (Figure 7) had a fibrous character unlike SP2 samples (Figure S3). Increasing the pH led to a decrease in fibrillar character and an increase in small clusters likely due to weak electrostatic interactions near the pI.^45^ Except for CF4, all samples with gellan gum or chitosan exhibited aggregation and clustering (Figure S3, Figure 7). CF4 aggregates exhibited some fibrillar characteristics similar to the observations made with AFM imaging (Figure 6). SP2 aggregates showed a smooth surface with some gaps within the matrix, but pH adjustment to 4 and 7 showed increased clustering similar to the fibrillated samples (Figure S3). Comparatively, GG2 formed a compact and uniform matrix likely due to electrostatic attraction, whereas CT2 showed increased gaps likely due to electrostatic repulsion between chitosan and soy protein at pH 2.^7,11,32^ GG4 aggregates showed defined clusters, while CT4 clusters had large uneven gaps, possibly due to electrostatic repulsion. SP7 and GG7 showed clustered surfaces, but CT7 appeared smoother with less clustering, likely due to chitosan–protein attraction at pH 7, similar to GG2.

**Figure 7:**
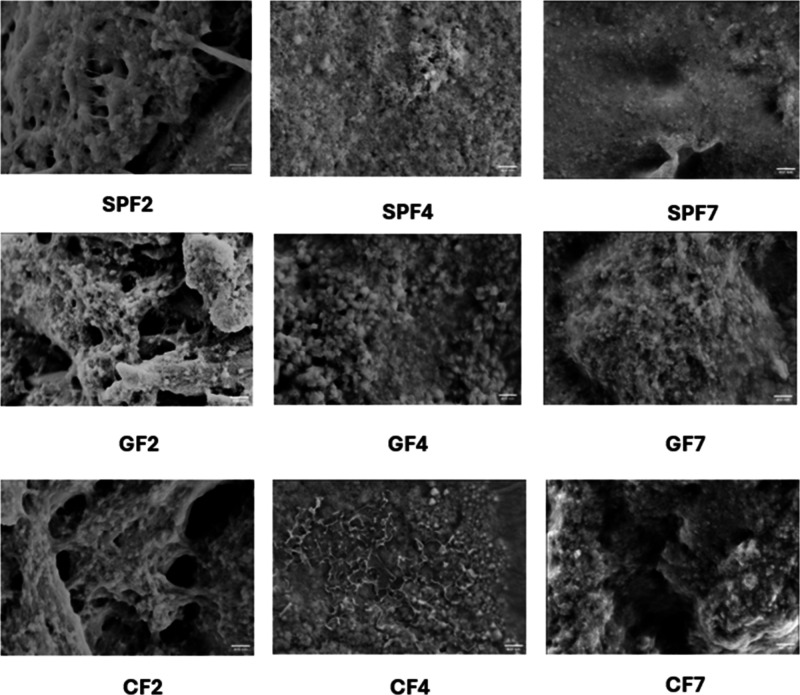
SEM images of fibrillated samples with and without addition of polysaccharides. Fibrillated soy protein at pH 2, 4, 7 = SPF2/4/7; fibrillated soy protein with gellan gum at pH 2, 4, 7 = GF2/4/7; fibrillated soy protein with chitosan at pH 2, 4, 7 = CF2/4/7, respectively. Scale bar = 400 nm. Magnification = 21K X.

### 3.8 Effects of pH and Polysaccharides on Flow Behavior of Protein Fibrils

Flow behavior of the samples under heating, cooling, and shear conditions was studied by using RVA. Among SP and SPF samples at different pH levels, only SPF2 showed gelation, indicated by an increase in viscosity during cooling (gel formation) and a drop in viscosity when shear was resumed (gel collapse) (Figure 8A, Table 2). This likely resulted from the conformation and structural rigidity of the fibrils, which undergo rearrangement and aggregation upon heating, facilitating stronger cross-linking and entanglement.^31,46^ Previous studies also report that fibrils can enhance gel strength in food systems.^46–48^ However, this was not observed for GF2 and CF2, suggesting that polysaccharides disrupted the protein–protein interactions needed for network formation.^3,49^ SP samples at all pH levels (2, 4, 7) showed no increase in viscosity during cooling or change after reapplication of shear, highlighting SPF2’s superior gelling capacity compared to unfibrillated soy protein. This gelling capacity was lost at pH 4 and 7, likely due to disruption of the fibrillar structure (Figure 6).

**Table 2:** Viscosity of Soy Protein Samples at Specific Time Points during the RVA Test

samples^a^	initial viscosity (cP)^b^	viscosity at the end of heating (cP)	viscosity at the end of cooling (cP)	viscosity after no shear hold (cP)	final viscosity (cP)
SP2	58.33 ± 7.09^a^	0.00 ± 2.00^a^	11.00 ± 6.56^b^	16.00 ± 8.72^b^	16.67 ± 5.13^b^
SPF2	21.67 ± 6.43^b^	4.33 ± 1.15^a^	71.00 ± 4.58^a^	93.67 ± 3.06^a^	82.33 ± 3.21^a^
SP4	15.67 ± 3.51^b^	1.00 ± 5.29^a^	14.33 ± 7.09^b^	17.33 ± 8.74^b^	21.33 ± 7.51^b^
SPF4	35.00 ± 4^b^	0.67 ± 3.51^a^	7.33 ± 4.16^b^	14.33 ± 3.51^b^	19.67 ± 8.96^b^
SP7	17.33 ± 6.35^b^	–1.00 ± 1.00^a^	8.33 ± 5.51^b^	13.00 ± 5.00^b^	14.00 ± 4.00^b^
SPF7	35.00 ± 15.62^b^	1.67 ± 2.89^a^	8.00 ± 3.61^b^	13.67 ± 1.53^b^	18.00 ± 1.00^b^
GG2	56.66 ± 6.51^a^	2.00 ± 4.00^b^	12.00 ± 6.56^b^	16.33 ± 6.03^c^	18.67 ± 7.09^b^
GF2	29.00 ± 4.58^cd^	6.33 ± 2.89^ab^	24.33 ± 12.66^b^	31.00 ± 14.18^c^	31.33 ± 14.01^b^
GG4	19.00 ± 1.73^d^	4.33 ± 2.08^b^	35.33 ± 4.04^b^	46.33 ± 4.73^c^	39.33 ± 2.52^b^
GF4	49.33 ± 8.02^ab^	11.67 ± 1.15^a^	22.67 ± 2.08^b^	25.33 ± 2.31^c^	26.67 ± 3.06^b^
GG7	38.00 ± 4^bc^	7.00 ± 2.65^ab^	199.67 ± 24.58^a^	365.33 ± 41.04^b^	189.00 ± 15.87^a^
GF7	47.33 ± 7.09^ab^	6.33 ± 0.58^ab^	216.00 ± 45.51^a^	688.67 ± 97.99^a^	221.33 ± 25.79^a^
CT2	101.33 ± 12.74^a^	5.33 ± 7.77^b^	17.33 ± 5.77^d^	23.00 ± 7.81^d^	24.67 ± 6.35^d^
CF2	48.67 ± 6.03^b^	9.00 ± 6.00^b^	18.33 ± 4.93^d^	25.00 ± 4.58^d^	27.33 ± 3.51^cd^
CT4	34.33 ± 4.93^b^	12.33 ± 3.06^b^	27.67 ± 2.08^cd^	34.33 ± 5.13^cd^	38.00 ± 7.94^bcd^
CF4	81.67 ± 21.59^a^	34.67 ± 3.51^a^	96.67 ± 3.79^a^	115.00 ± 8.89^a^	93.67 ± 6.66^a^
CT7	19.82 ± 1.91^b^	18.37 ± 4.15^b^	64.28 ± 3.87^b^	71.53 ± 4.39^b^	43.98 ± 5.21^b^
CF7	32.00 ± 7.94^b^	16.00 ± 2.65^b^	35.00 ± 5.00^c^	42.33 ± 4.16^c^	41.00 ± 1.73^bc^

a Unfibrillated soy protein at pH 2, 4, 7 = SP2, 4, 7; unfibrillated soy protein with gellan gum at pH 2, 4, 7 = GG2, 4, 7; unfibrillated soy protein with chitosan at pH 2, 4, 7 = CT2, 4, 7, respectively. Fibrillated soy protein at pH 2, 4, 7 = SPF2, 4, 7; fibrillated soy protein with gellan gum at pH 2, 4, 7 = GF2, 4, 7; fibrillated soy protein with chitosan at pH 2, 4, 7 = CF2, 4, 7, respectively.

b Lowercase letters denote significant differences in viscosity in SP and SPF, GG and GF, and CT and CF samples at a specific time point (*p* < 0.05).

**Figure 8:**
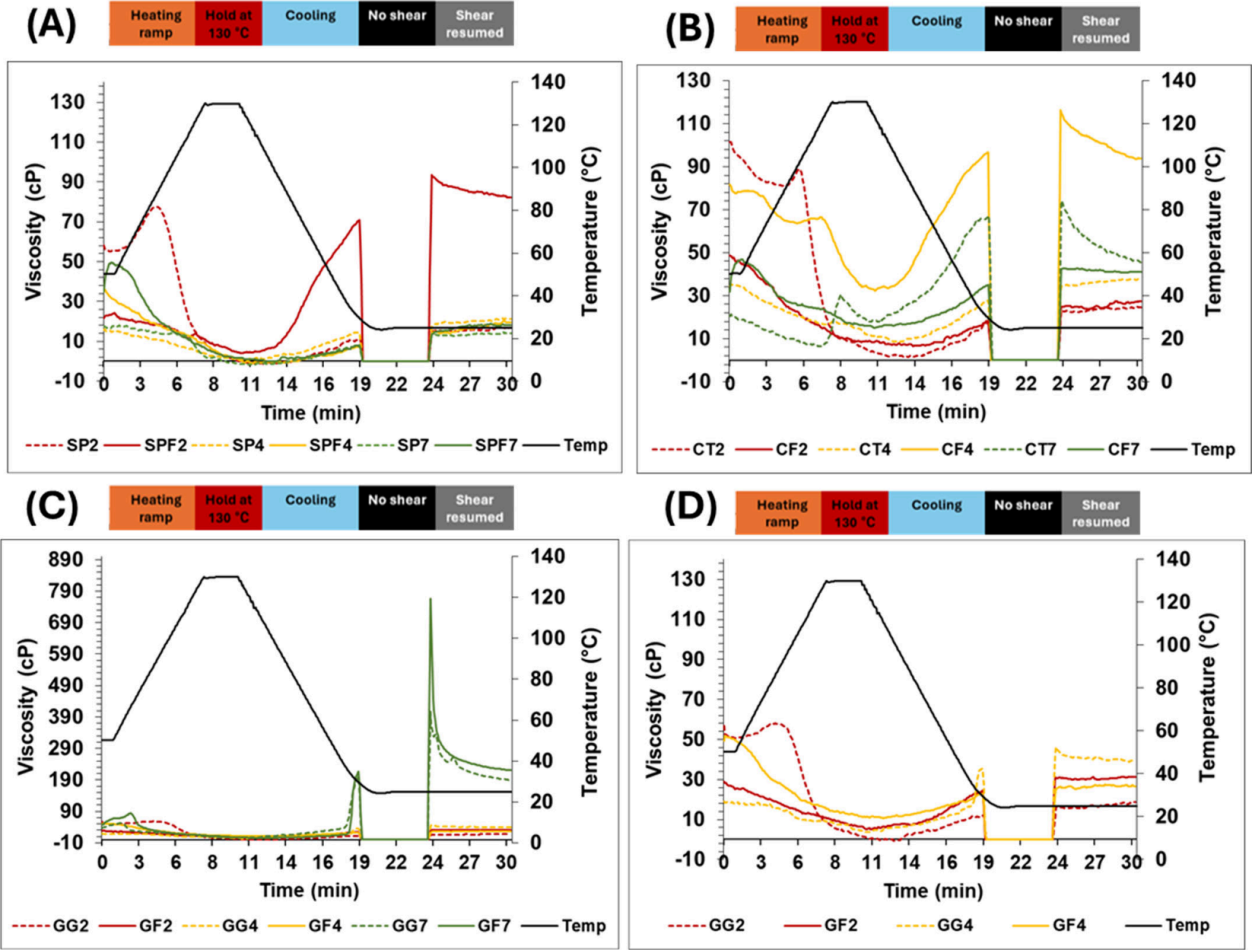
Flow behavior of different samples measured using the RVA. (A) Unfibrillated and fibrillated soy protein at pH 2, 4, 7 = SP2/4/7 and SPF2/4/7, respectively; (B) unfibrillated and fibrillated soy protein with chitosan at pH 2, 4, 7 = CT2/4/7 and CF2/4/7, respectively; (C) unfibrillated and fibrillated soy protein with gellan gum at pH 2, 4, 7 = GG2/4/7 and GF2/4/7, respectively. (D) Magnified view of the gellan gum–treatment graph to better visualize the GG2, GG4, GF2, and GF4 samples.

GG7 and GF7 showed a substantial increase in viscosity, surpassing SPF2 and indicating strong gel formation (Figure 8C,D). This could be due to electrostatic repulsion at pH 7 between like-charged gellan gum and soy protein, leading to individual biopolymer dissociation. Thus, gellan gum could have formed an independent gel network, creating a double-network system where protein and polysaccharide were excluded from each other but still produced a uniform gel due to heating under constant shear.^50,51^ GF7 showed even higher viscosity than GG7 after the ‘no shear’ phase (Figure 8C, Table 2), indicating that remnant fibrils in GF7 reinforced the gel, despite aggregation and structural loss observed by AFM and particle size analysis (Figure 1A, Figure 6). This aligns with FTIR data showing preserved antiparallel β-sheets in GF7 and supports the idea that aggregation can retain some fibrillar structure (Section 3.5, Table 1). Among CT and CF samples, CF4 showed viscosity trends most comparable to those of SPF2 (Figure 8B, Table 2). This is consistent with earlier results (Sections 3.1 and 3.8), confirming that chitosan at pH 4 best preserved the fibril structure and, as a result, its functionality.

### 3.9 Effects of pH and Polysaccharides on Phase Stability

Phase stability was visually examined on samples pre- and post-RVA treatment after overnight storage. Fibrillated samples across all pH levels and polysaccharide additions showed better phase stability than their unfibrillated counterparts (Figure 9). GF2 and CF2 samples maintained a single phase similar to that of SPF2, suggesting that gellan gum and chitosan did not disrupt fibril phase stability. Even post-RVA, GF2 and CF2 remained single-phase, likely because of heat treatment causing protein unfolding then refolding, allowing recovery of fibril secondary structure upon cooling.^52^

**Figure 9:**
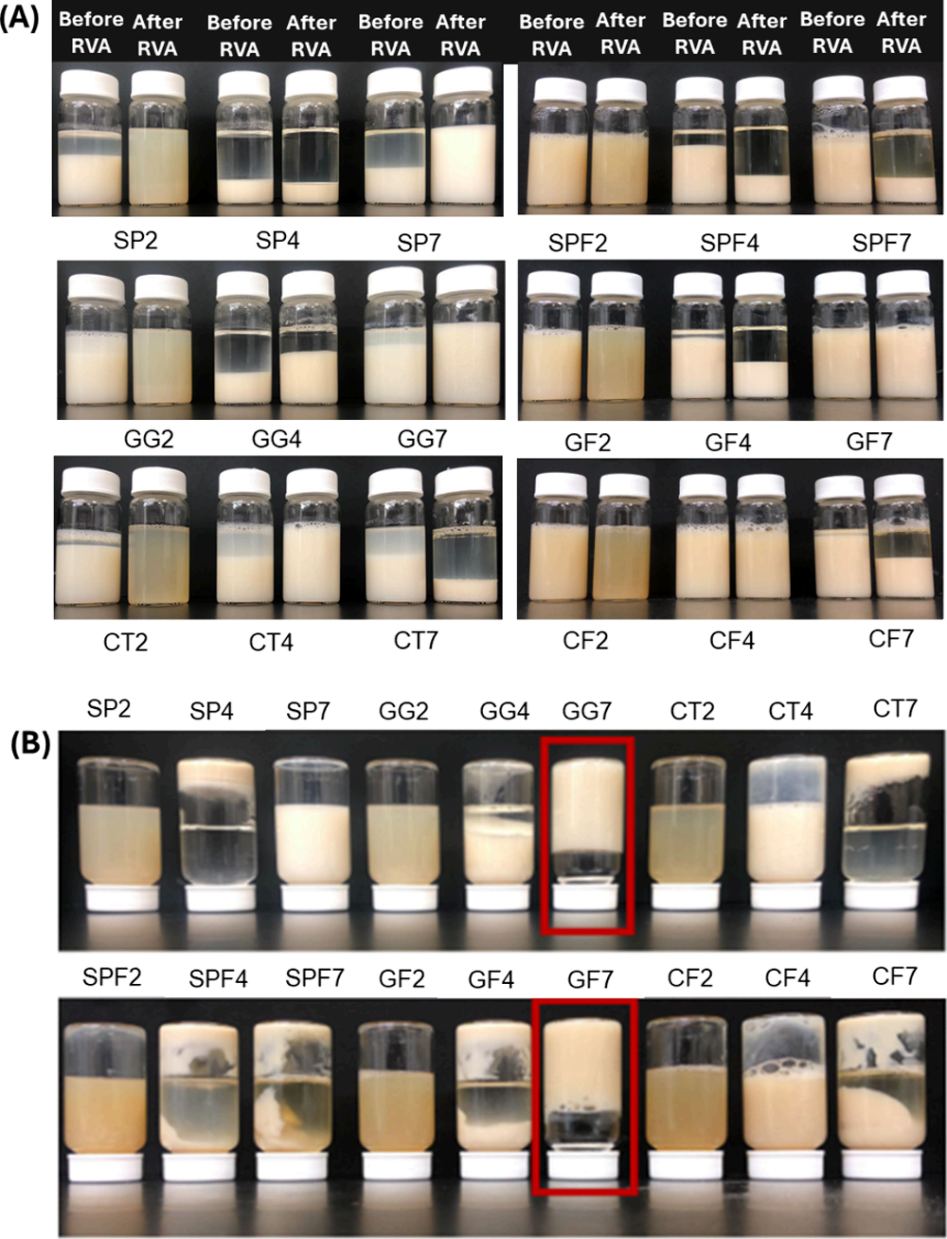
(A) Samples before and after RVA treatment analyzed for phase separation after overnight storage in refrigerated conditions. (B) Inversion of samples stored overnight after RVA treatment to assess the formation of self-supporting gels. Unfibrillated soy protein at pH 2, 4, 7 = SP2/4/7; unfibrillated soy protein with gellan gum at pH 2, 4, 7 = GG2/4/7; unfibrillated soy protein with chitosan at pH 2, 4, 7 = CT2/4/7, respectively. Fibrillated soy protein at pH 2, 4, 7 = SPF2/4/7; fibrillated soy protein with gellan gum at pH 2, 4, 7 = GF2/4/7; fibrillated soy protein with chitosan at pH 2, 4, 7 = CF2/4/7, respectively.

At pH 4, only CF4 remained single-phase, likely due to electrostatic repulsion and minimal aggregation (Figure 9).^11^ RVA processing exacerbated phase instability with only CF4 retaining a single-phase, similar to SPF2. GF7 also remained stable, likely due to electrostatic repulsion between gellan gum and soy protein.^11^ In contrast, SPF7 and CF7 showed slight phase separation, which worsened after RVA processing, suggesting that fibril structure disruption also reduced the system’s ability to maintain phase stability after thermal processing. Similar trends were observed for SPF4 and GF4. After inverting the RVA-treated samples, only GG7 and GF7 formed self-supporting gels (Figure 9B), aligning with the RVA trends (Section 3.9, Figure 8).

Thus, complexation with ionic polysaccharides was found to significantly influence the fibril structure and functionality in response to pH changes. Complexation with chitosan at pH 4 effectively minimized aggregation and loss of fibril β-sheet structure as confirmed through particle size analysis, FTIR, and AFM imaging (Figure 1A, Table 1, Figure 6). This combination also maintained fibril functionality and phase stability after exposure to elevated temperatures (Sections 3.8 and 3.9). As a result, the sample demonstrated excellent phase stability and gelling capacity, supporting its potential use in matrices such as beverages and yogurts. Future studies can explore the potential of fibrils as delivery agents in these matrices. In contrast, complexation of fibrils with gellan gum did not optimally preserve the fibrillar structure upon changing pH. However, at pH 7, the gellan gum–fibril complexes formed a self-supporting gel. Notably, the fibrillated samples with gellan gum at pH 7 demonstrated superior gelling properties compared to both the fibrils at pH 2 and its unfibrillated counterpart at pH 7, highlighting the synergistic benefits of fibrillation and gellan gum addition (Section 3.9, Figure 9B). However, the exact mechanism behind the increased gel strength in fibrillated samples compared to unfibrillated remains unclear, presenting an interesting avenue for future research. Based on these findings, our follow-up work will incorporate techniques such as rheometry, confocal microscopy, and differential scanning calorimetry (DSC) to better characterize the gelation mechanism of fibrils and their complexes.

## Supplementary Material



## Notes

FTIR spectra of all samples, AFM and SEM images of unfibrillated samples (PDF)

## Abbreviations

SP, GG, CT, SPF, GF, and CF: soy protein, soy protein with gellan gum, soy protein with chitosan, soy protein fibrils, soy protein fibrils with gellan gum, and soy protein fibrils with chitosan, respectively
ThT: Thioflavin T
H_0_: surface hydrophobicity
ATR-FTIR: attenuated total reflectance Fourier-transform infrared spectroscopy
AFM: atomic force microscopy
SEM: scanning electron microscopy
