# Supplementary material for: Complexation with Ionic Polysaccharides Mitigates pH-Dependent Degradation of Soy Protein Fibril Structure and Functionality
Source: J Agric Food Chem. 2025 Aug 18;73(36):22734–45. doi: 10.1021/acs.jafc.5c07627 (PMC12503362; doi:10.1021/acs.jafc.5c07627)
Supplement: Supplementary file 1 [file jf5c07627_si_001.pdf]

Supporting information

Complexation with ionic polysaccharides mitigates pH-dependent degradation of soy protein fibril structure and functionality

Sanjana Sawant [a], and Audrey L. Girard [a,*]

[a] Department of Food Science, University of Wisconsin-Madison, Madison, WI 53706, USA

*Corresponding author: Email algirard@wisc.edu; Phone +1 608-890-4877; Fax +1 608-262-6872

(A)

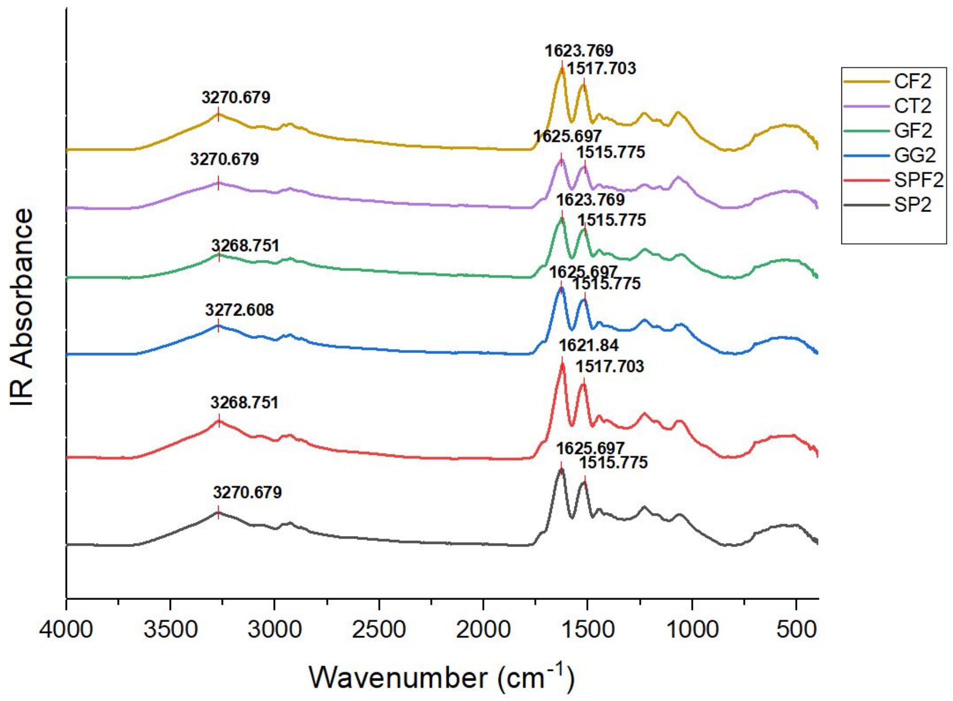

1623.769
1517.703
3270.679
1625.697
1515.775
3270.679
1623.769
1515.775
3268.751
1625.697
1515.775
3272.608
1621.84
1517.703
3268.751
1625.697
1515.775
3270.679
CF2
CT2
GF2
GG2
SPF2
SP2
IR Absorbance
4000
3500
3000
2500
2000
1500
1000
500
Wavenumber (cm^{-1})

(B)

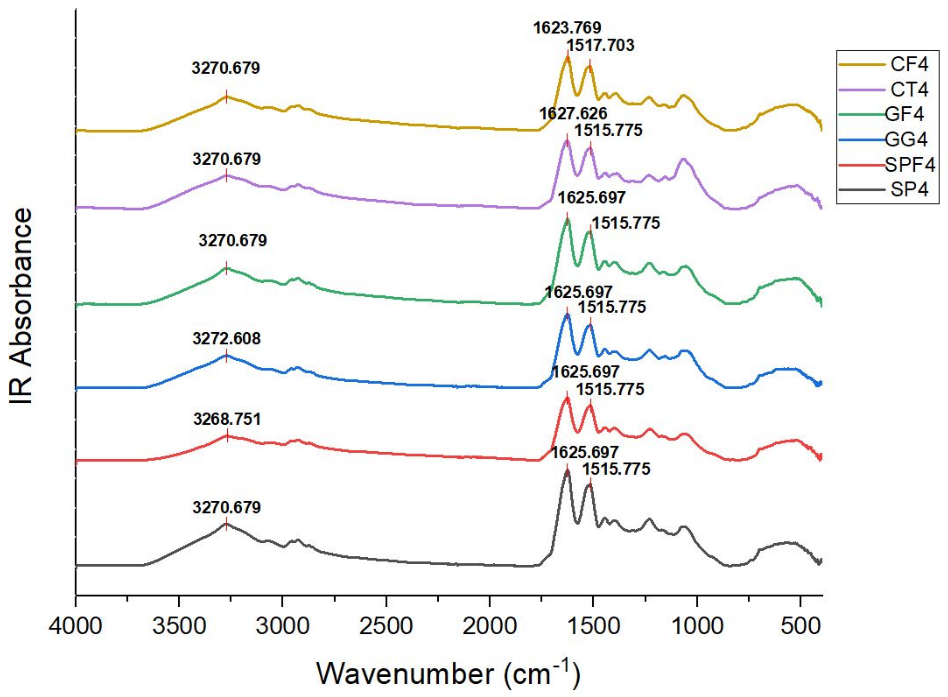

1623.769
1517.703
3270.679
1627.626
1515.775
3270.679
1625.697
1515.775
3270.679
1625.697
1515.775
3272.608
1625.697
1515.775
3268.751
1625.697
1515.775
3270.679
CF4
CT4
GF4
GG4
SPF4
SP4
IR Absorbance
4000
3500
3000
2500
2000
1500
1000
500
Wavenumber (cm^{-1})

(C)

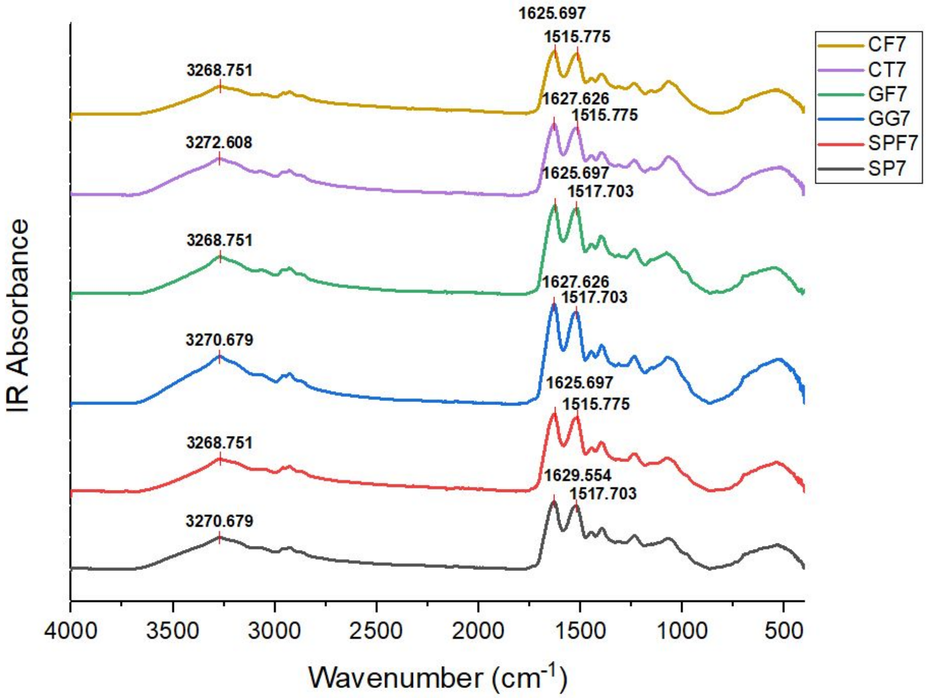

Fig S1. FTIR Spectra of different samples (Un-fibrillated soy protein at pH 2,4,7 – SP 2, 4, 7; un-fibrillated soy protein with gellan gum at pH 2, 4, 7 – GG2, 4, 7; un-fibrillated soy protein with chitosan at pH 2, 4, 7 – CT2, 4, 7, respectively. Fibrillated soy protein at pH 2,4,7 – SPF 2, 4, 7; fibrillated soy protein with gellan gum at pH 2, 4, 7 – GF2, 4, 7; fibrillated soy protein with chitosan at pH 2, 4, 7 – CF2, 4, 7, respectively). (A) Samples at pH 2, (B) Samples at pH 4, and (C) Samples at pH 7.

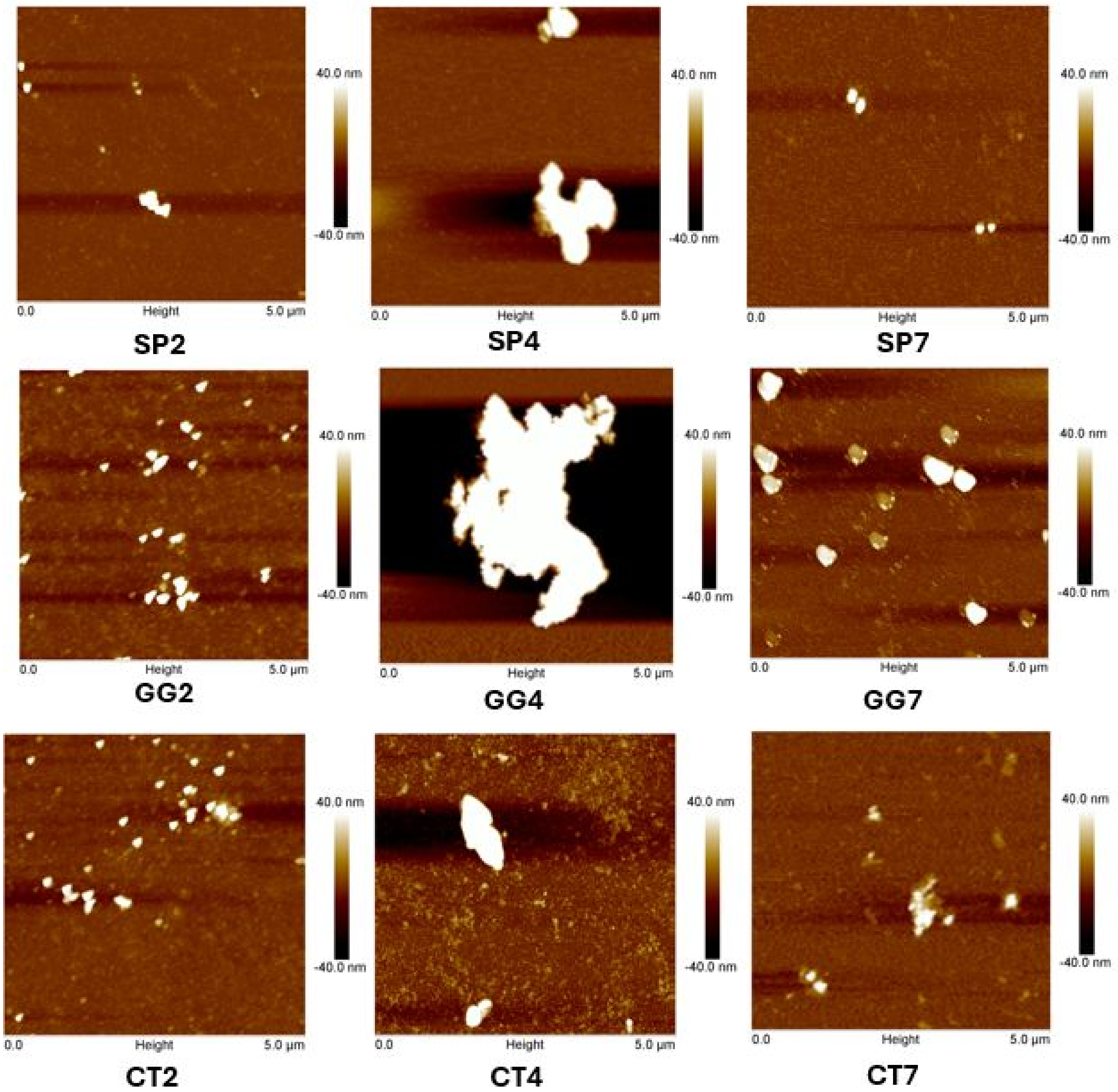

Fig S2. AFM images of un-fibrillated samples with and without addition of polysaccharides. (Un-fibrillated soy protein at pH 2,4,7 – SP 2, 4, 7; un-fibrillated soy protein with gellan gum at pH 2, 4, 7 – GG2, 4, 7; un-fibrillated soy protein with chitosan at pH 2, 4, 7 – CT2, 4, 7, respectively).

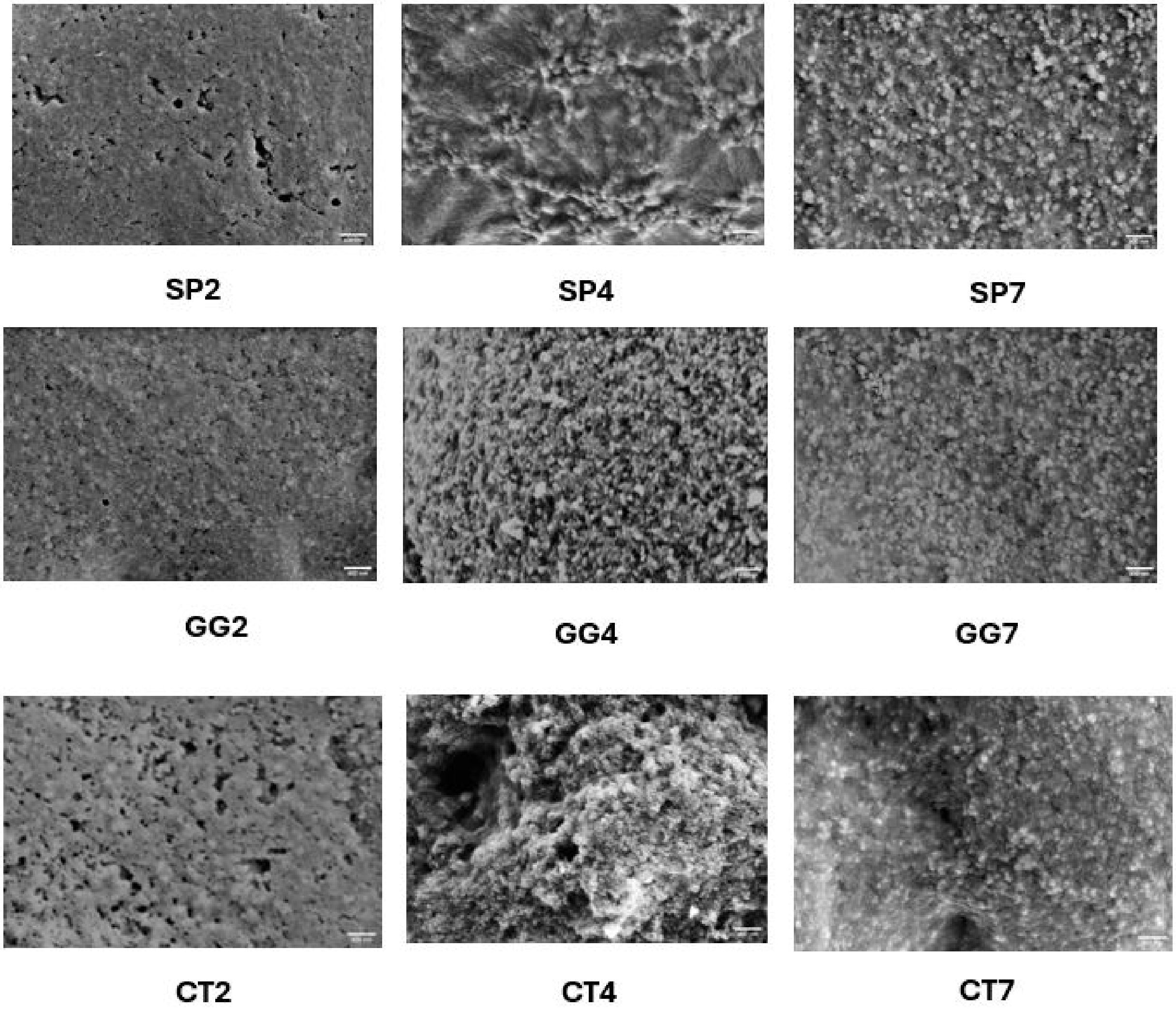

Fig S3. SEM images of un-fibrillated samples with and without addition of polysaccharides. (Un-fibrillated soy protein at pH 2,4,7 – SP 2, 4, 7; un-fibrillated soy protein with gellan gum at pH 2, 4, 7 – GG2, 4, 7; un-fibrillated soy protein with chitosan at pH 2, 4, 7 – CT2, 4, 7, respectively).
